# Egg Reintroduction Following Oral Food Challenge in Japanese Children

**DOI:** 10.3389/falgy.2021.618713

**Published:** 2021-04-01

**Authors:** Hiroki Masumi, Yutaka Takemura, Tomoyuki Arima, Koji Yamasaki, Megumi Nagai, Norihiro Inoue, Keisuke Sugimoto

**Affiliations:** ^1^Department of Pediatrics, Kaizuka City Hospital, Kaizuka, Japan; ^2^Department of Pediatrics, Kindai University Faculty of Medicine, Osakasayama, Japan; ^3^Department of Pediatrics, National Hospital Organization Osaka Minami Medical Center, Kawachinagano, Japan

**Keywords:** egg allergy, challenge test, children, Japanese, food hypersensitivity

## Abstract

**Background:** Oral food challenge (OFC) is the most reliable method for diagnosing food allergies. However, the scarcity of long-term data on eating habits of people after a negative OFC poses a challenge for provisional medical care.

**Objective:** This study was performed to investigate the percentage of people who could reintroduce eggs into their diet several years after an OFC.

**Methods:** Study participants included 0–6-year-old children with negative results from an OFC using one egg as the food allergen, boiled for 20 min, from January 2012–March 2017, 1–3 years after the OFC.

**Results:** A total of 72 subjects were analyzed, out of which 52 were males (72.2%). The median age (range) was 20 months (16–32.3), and the median age (range) at the first OFC was 15 months (12.8–23.3). Eggs were reintroduced in 62 cases (86.1%), while 10 cases (13.9%) did not undergo any diet change. The adjusted odds ratio (OR, 95% CI), with post-OFC to pre-OFC anxiety ≥ 0.2, was 9.4 (1.0–86), *p* = 0.04; OR for allergic symptoms that occurred post-OFC was 2.2 (0.45–11), *p* = 0.34; OR for initial OFC at an age of ≥15 months was 3.2 (0.54–19), *p* = 0.2; and OR for the history of anaphylaxis from eggs was 0.17 (0.02–1.5), *p* = 0.11.

**Conclusion:** Most cases reintroduced eggs after an OFC. However, reintroduction did not occur in some cases, which was associated with greater anxiety among caregivers post-OFC. If the caregiver's anxiety is intense, it is necessary to provide psychological intervention and dietary guidance when reintroducing eggs at home after an OFC and to follow-up outpatient long-term progress.

## Introduction

Food allergies (FAs) are common in children, with a prevalence reported to be 5% or less (1). However, prevalence rates have been recently rising (2), making FA a major health concern in children. Among Japanese children, eggs are the most common food allergen, followed by milk and wheat (3).

The most reliable method for diagnosing an egg allergy is an oral food challenge (OFC) (4). Generally, an OFC is performed according to a protocol set by each facility. The expected total allergen load in an OFC takes into account risk factors such as medical history, immunological test results, food type, presence or absence of underlying disease, and age. A higher load is set for low-risk individuals and a lower load for high-risk individuals. Normally, the highest expected total allergen load in an egg OFC is one egg, which is regularly consumed by non-allergic children.

However, even if an OFC is assessed as negative because no allergic symptoms were induced from eating one egg, the subject cannot be deemed to be allergy-free based only on this result. It is then necessary to confirm the absence of allergic symptoms by having the subject consume the allergen multiple times at home. Even with a negative OFC result, sometimes allergic symptoms that require treatment occur when the allergen is consumed at home (5). A follow-up survey after OFC found that even with a negative result, 13.2% of subjects did not reintroduce eggs (6). It is not easy to start eating (reintroduce) the same food as non-allergic children do when medical professionals or caregivers have been blocking its consumption for a period of time. Factors that inhibit reintroduction include the occurrence of allergic symptoms at home after an OFC, being female, a long period of food exclusion from the diet, and anxiety or fear among caregivers (6–9). A history of anaphylaxis and high levels of egg white-specific IgE antibodies (sIgE) reportedly inhibit the acquisition of egg allergy resistance and induce severe allergic symptoms from an egg OFC (10, 11). If a child who has been restricted from eating eggs for a certain period is allowed to begin eating them after an egg OFC, care needs to be provided so eggs can be reintroduced safely and effectively. This practice includes consideration of whether a history of anaphylaxis from eggs or high levels of egg white-sIgE exists, as well as other factors that inhibit reintroduction.

However, there have been no studies on reintroduction after the initial OFC in Japan. Therefore, in this study, we investigated the percentage of patients who reintroduced eggs at home after an egg OFC and clarified the factors associated with not reintroducing eggs. Improving our understanding of these factors will help identify the patient group that medical professionals should focus on when reintroducing eggs at home and will be useful when providing guidance to egg allergy patients in the future.

## Materials and Methods

### Study Design and Participants

This was a cross-sectional study. This study was conducted at two facilities, at the pediatric departments of Kindai University Hospital and Kaizuka City Hospital.

Study participants were those who met all selection criteria and did not fall under any exclusion criteria. The selection criteria were (1) OFC performed at 0–6 years of age, (2) negative egg OFC results (STEP 3), and (3) 1–3 years had passed since the initial OFC. The exclusion criteria were (1) duration of ≥6 months from egg white-sIgE measurement to the initial OFC, (2) blank or severely deficient questionnaire entries, and (3) not returning the questionnaire.

### Questionnaire and Survey Form

A letter containing the questionnaire was sent to all study participants in August 2018. The deadline for its return was 1 month later. The questionnaire included parents' ages, presence or absence of an allergy history for the study participant (FAs other than egg allergy, atopic dermatitis, bronchial asthma), presence or absence of allergies in the family (FAs, atopic dermatitis, bronchial asthma), presence or absence and the number of siblings, whether the subject was treated the same as children without egg allergies at preschool or elementary school events involving food, whether they ate one or more cooked or raw eggs per week, the duration from an OFC with 30–40 g of cooked egg white to the reintroduction of eggs, presence or absence of allergic symptoms after an OFC with 30–40 g of cooked egg white, need for treatment by a medical institution when symptoms occurred and content of this treatment, and degree of anxiety before and after egg OFC [0–10 on a visual analog scale (VAS) scale].

In addition, investigators filled out survey forms by checking the necessary factors from the subjects' medical records. These included sex, date of birth, egg white- and ovomucoid-sIgE (ImmunoCAP®), wheal diameter in the skin-prick test (SPT), presence or absence of allergic symptoms or anaphylaxis episodes before an egg OFC, possession of self-injectable adrenaline, age at first egg OFC, treatment history during previous egg OFC, and purpose of egg OFC.

### OFC Objective

The purposes of the OFC we conducted were as follows and were created based on the Japanese Food Allergy Guidelines (4).

① The OFC was performed to determine why a blood test showed elevated egg-sIgE despite no history of egg consumption.”
② OFC was performed for diagnosis of subjects showing symptoms that suggest allergies after eating eggs but are not symptoms related to evident FAs, such as mild skin symptoms and symptoms occurring after a prolonged time from eating to their onset.
③ For study participants previously diagnosed with an egg allergy, the test was conducted to assess symptom threshold, determine the amount that can be consumed safely, and confirm resistance acquisition.

In scenarios (1) and (2), an OFC was performed for diagnostic purposes to test for an egg allergy. In situation (3), an OFC was performed for reasons other than diagnostic purposes.

### OFC Method

Here, the food challenge in the egg OFC was an egg white boiled for 20 min. Steps 1–3 were determined based on the expected total intake. Each step was selected according to the purpose of OFC and egg white-sIgE. The expected total intake, which corresponds to the daily intake, is step 3, in which about 30–40 g (one egg) was consumed at once or divided into 2–3 portions. For OFC with diagnostic purposes, OFC of the step 3 was performed when egg white-sIgE was 0 to <3.5 UA/mL. For OFC related to other purposes, the physician in charge determined the total amount for the step based on whether allergic symptoms were induced and on the severity of symptoms if any occurred. If step 1 or 2 OFC was negative, the patient was allowed to eat eggs at home, with the total amount consumed as the upper limit. Later, each dose of OFC was increased; if the OFC in step 3 was negative, one heated chicken egg was ingested multiple times at home, and it was confirmed that there were no allergic symptoms. Thereafter, the amount of egg protein using one chicken egg was set as the upper limit, the intake of egg dishes with a weakened degree of heating was instructed, and the patient's eating status was confirmed through outpatient care.

### OFC Result Criteria and Severity of Positives

The OFC result criteria were positive if obvious allergic symptoms were induced within 2 h after the final load and negative if not. Transient skin symptoms appearing only around the oral cavity; itching and discomfort in the oral cavity and lip swelling appearing transiently; and itching and discomfort in the pharynx appearing transiently were not considered obvious allergic symptoms and were put on hold. If the decision was put on hold, the same OFC was carried out at a later date. For the severity of OFC-positive individuals, modified Sampson classification (12) was used except for the symptoms put on hold (Table 1).

**Table 1:** Severity classification of induced symptoms in people positive for the oral food challenge (modified Sampson classification).

**Grade**	**Skin**	**GI tract**	**Respiratory tract**	**Cardiovascular**	**Neurological**
1	Localized persistent flushing other than around oral cavity, and urticaria, angioedema^*^	Oral pruritus, oral “tingling,” mild lip swelling^†^	Pharynx pruritus, pharynx tingling^†^	–	–
2	Generalized pruritus, flushing, urticaria, angioedema	Nausea and/or emesis, diarrhea, transient abdominal pain	Nasal congestion and/or sneezing	–	Change in activity level
3	Any of the above	Repetitive vomiting and diarrhea, persistent abdominal pain	Rhinorrhea, marked congestion, sensation of throat pruritus or tightness	Tachycardia (increase > 15 beats/min)	Change in activity level plus anxiety
4	Any of the above	Any of the above	Hoarseness, “barky” cough, difficulty swallowing, dyspnea, wheezing, cyanosis	Dysrhythmia and/or mild hypotension	“Light headedness,” feeling of “pending doom”
5	Any of the above	Any of the above	Respiratory arrest	Severe bradycardia and/or hypotension or cardiac arrest	Loss of consciousness

* *The transient appearance of skin symptoms around the oral cavity, which corresponds to the skin symptom grade 1 of the Sampson classification, was judged to be pending*.

† *Among the symptoms corresponding to Grade 1 of the Sampson classification that appeared transiently was judged to be pending*.

### Outcomes

Primary outcome: Percentage of subjects who reintroduced eggs 1–3 years after step 3, egg OFC.

Secondary outcomes:

1) Factors associated with not reintroducing eggs
2) Duration until the reintroduction of eggs

The complete reintroduction of eggs for this study was defined as the study participant having eaten at least one egg per week during regular meals at home or when the study participant was treated the same way as children without egg allergies at preschool or elementary school events involving food.

### Statistical Analysis

Statistical analyses were performed using the statistical software Easy R (EZR) version 2.4. Fisher's exact test was used to analyze the presence or absence of egg reintroduction and various other factors. Caregivers' anxiety regarding OFC was calculated by dividing post-OFC anxiety by pre-OFC anxiety (anxiety level = post-OFC anxiety score/pre-OFC anxiety score). In the analysis, the continuous explanatory variables (age in months, age in months at first OFC, father's/mother's age, egg white-sIgE, ovomucoid-sIgE, SPT, and anxiety level) were divided into high and low levels based on the median value and subsequently analyzed as binary variables.

In addition, factors that exhibited *p* < 0.2 in univariate analyses were used in the logistic regression analysis of factors related to the inability to reintroduce eggs. *p* < 0.05 was considered a statistically significant difference.

### Ethical Considerations

This study was approved by the ethics committees of the Kindai University Faculty of Medicine (approval No. 29-215) and Kaizuka City Hospital (approval No. 168). To obtain consent for using personal information, the content of the study was posted on the hospitals' websites, and not sending the questionnaire back was considered a refusal to participate.

## Results

### Study Participants

**Figure 1:**
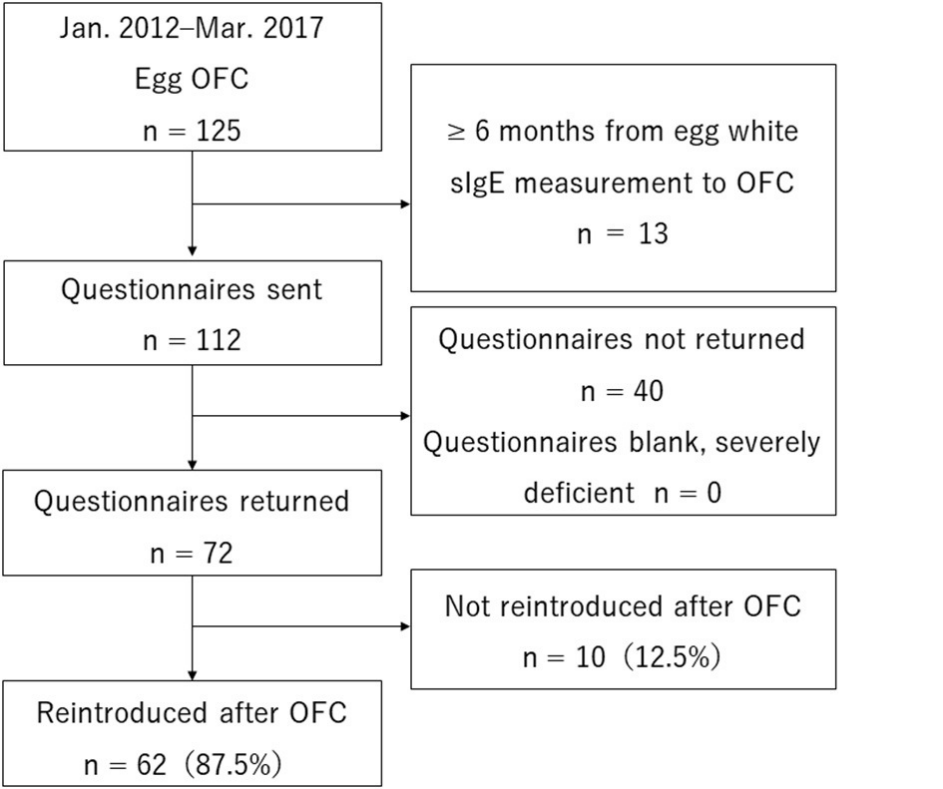
Subjects.

A total of 125 people underwent step 3, egg OFC, and met the selection criteria. Of these, 13 were excluded for meeting one of the exclusion criteria (6 months from sIgE measurement to OFC). Questionnaires were sent to 112 people. None were excluded for being sent back blank or severely deficient. Overall, 72 people (64%) sent back the questionnaire and were enrolled in the study (Figure 1).

### Study Participant Characteristics

Table 2 shows the study participants' characteristics. There were 52 boys (72.2%), and their median age (range) was 20 months (16–32.3). The median age at the first OFC was 15 months (12.8–23.3), and 55 people (76.4%) underwent an OFC for diagnostic purposes. Prior to the OFC, 14 people (19.4%) had a history of anaphylaxis from eating eggs, and 19 people (26.4%) had allergic symptoms from eating eggs at home following an OFC. Furthermore, 16 people (23.5%) had concomitant bronchial asthma, and 48 people (69.6%) had a family member with a history of allergies. The median sIgE (UA/mL, range) was 7.9 (4.3–13.9) for egg whites and 2.0 (0.4–6.0) for ovomucoid. The median SPT wheal diameter (mm, range) was 8 (5–9.5). The ratio (range) of post-OFC to pre-OFC anxiety was 0.2 (0–0.5).

**Table 2:** Subject characteristics.

	***n* = 72**
Sex: male (%)	52 (72.2)
Median age in months (range)	20 (16–32.3)
Median age in months at first OFC (range)	15 (12.8–23.3)
**Median age of parents (range)**	
Father^*^	37 (33–41.5)
Mother^†^	35.5 (32–40)
**Advanced education (university graduate)**	
Father (%)^‡^	38 (57.6)
Mother (%)^‡^	38 (57.6)
Mother in the workforce (%)^†^	49 (72.1)
Has siblings (%)^§^	55 (79.7)
OFC purpose: diagnostic (%)	55 (76.4)
History of anaphylaxis from eating eggs (%)	14 (19.4)
Allergic symptoms induced at home after OFC (%)	19 (26.4)
Has BA (%)^†^	16 (23.5)
Family history of allergies (%)^§^	48 (69.6)
Egg white-sIgE (UA/mL, range)	7.9 (4.3–13.9)
Ovomucoid-sIgE (UA/mL, range)^||^	2.0 (0.4–6.0)
Mean SPT wheal diameter (mm, range)^^*^^	8 (5–9.5)
Ratio of post-OFC to pre-OFC anxiety (range)	0.2 (0–0.5)

*OFC, Oral food challenge; BA, Bronchitic asthma; sIgE, specific IgE antibody; SPT, Skin-Prick Test*.

* *Data missing for 5 cases*.

† *Data missing for 4 cases*.

‡ *Data missing for 6 cases*.

§ *Data missing for 3 cases*.

|| *Data missing for 1 case*.

### Percentage Reintroduced and Time Until Reintroduction

Of the 72 subjects, 62 (86.1%) were able to reintroduce eggs, while 10 (13.9%) were not (Figure 1). The basis for determining non-reintroduction was that the study participant was not eating at least one whole egg per week during regular meals at home in eight subjects (11.1%) and that the study participant was not treated the same as other children without egg allergies during preschool or elementary school events involving food in two subjects (2.8%).

The median time (range) until eggs were reintroduced after OFC was 6 months (3–12 months). The reason why eggs were not reintroduced was a matter of preference in six subjects, as allergic symptoms occurred at home after step 3 OFC in three subjects, and for another reason in one subject. None of the three subjects who experienced allergic symptoms at home needed examination at a medical institution or took medication.

### Factors Associated With Reintroducing Eggs

Univariate analyses were performed to investigate factors associated with not reintroducing eggs (Table 3). The OR (95% CI) with the occurrence of allergic symptoms after an OFC was 3.4 (0.7–17), *p* = 0.12. The OR (95% CI) with the first OFC at ≥15 months of age was 3.7 (0.7–38), *p* = 0.17. The OR (95% CI) with a ≥0.2 ratio of post-OFC to pre-OFC anxiety was 10.6 (1.3–491), *p* = 0.014. The OR (95% CI) with history of anaphylaxis was 0.12 (0.01–1.1), *p* = 0.03.

**Table 3:** Factors associated with not reintroducing eggs.

**Eggs reintroduced after OFC**			**Unadjusted**		**Adjusted**	
	**No**	**Yes**	**OR (95% CI)**	***p*-value**	**OR (95% CI)**	***p*-value**
Sex: male (%)	5 (50)	41 (66)	0.51 (0.1–2.5)	0.48	–	–
Median age in months (≧20 months) (%)	6 (60)	30 (48)	1.6 (0.34–8.4)	0.74	–	–
Median age in months at first OFC (≧15 months) (%)	8 (80)	32 (52)	3.7 (0.7–38)	0.17	3.2 (0.54–19)	0.2
**Median age of parents (%)**						
Father (≧37 years old)^*^	6 (60)	28 (49)	1.5 (0.3–8.3)	0.73	–	–
Mother (≧35 years old)^†^	4 (40)	33 (57)	0.5 (0.1–2.4)	0.49		
**Advanced education (university graduate)**						
Father (%)^‡^	5 (50)	28 (50)	1 (0.2–4.9)	1	–	–
Mother (%)	5 (50)	28 (50)	1 (0.2–4.9)	1		
Mother in workforce (%)^†^	8 (80)	39 (67)	1.9 (0.3–20)	0.7	–	–
Has siblings (%)^§^	8 (80)	45 (76)	1.2 (0.2–13)	1	–	–
OFC purpose: diagnostic (%)	6 (60)	46 (74)	1.9 (0.3–9.3)	0.45	–	–
History of anaphylaxis (%)	3 (30)	3 (4.8)	0.12 (0.01–1.1)	0.03	0.17 (0.02–1.5)	0.11
Allergic symptoms induced at home after OFC (%)	5 (50)	14 (23)	3.4 (0.7–17)	0.12	2.2 (0.45–11)	0.34
Has BA (%)^†^	1 (11)	6 (10)	1.1 (0.02–11)	1	–	–
Family history of allergies (%)^§^	8 (80)	40(68)	0.53 (0.1–3.0)	0.7	–	–
Egg white-sIgE (≧7.9) (%)	6 (60)	32 (52)	1.4 (0.3–7.4)	0.74	–	–
Ovomucoid-sIgE (≧2.0) (%)^||^	6 (67)	29 (47)	2.2 (0.4–15)	0.48		
Mean SPT wheal diameter (≧8mm) (%)^^*^^	6 (60)	37 (65)	0.8 (0.2–4.4)	0.74	–	–
Ratio of post-OFC to pre-OFC anxiety (≧0.2) (%)	9 (90)	28 (45)	10.6 (1.3–491)	0.014	9.4 (1.0–86)	0.04

* *Data missing for 5 cases*.

† *Data missing for 4 cases*.

‡ *Data missing for 6 cases*.

§ *Data missing for 3 cases*.

|| *Data missing for 1 case*.

In logistic regression analysis, the adjusted OR (95% CI) with allergic symptoms induced by eating eggs at home after OFC was 2.2 (0.45–11), *p* = 0.34. The adjusted OR (95% CI) at an age of ≥15 months at the first OFC was 3.2 (0.54–19), *p* = 0.2. The adjusted OR (95% CI) with a ≥0.2 ratio of post-OFC over pre-OFC anxiety was 9.4 (1.0–86), *p* = 0.04. The adjusted OR (95% CI) with a history of anaphylaxis was 0.17 (0.02–1.5), *p* = 0.11 (Table 3).

## Discussion

This study was the first to examine egg consumption after egg OFC among Japanese children. Over 80% of Japanese children who were negative in step 3 egg OFC were able to reintroduce eggs 1–3 years after the OFC. This result is not substantially different from the reported percentage of those who reintroduced eggs (6). Nevertheless, some children were unable to reintroduce eggs into their diet. The anxiety some caregivers felt about feeding eggs to their children after an OFC hampered the reintroduction. Other factors that delayed or hindered the reintroduction of eggs included a higher age at the first OFC, the occurrence of allergic symptoms after eating eggs at home after the OFC, and having a history of anaphylaxis due to eggs prior to the OFC.

The reintroduction rates following an OFC for peanuts and hazelnuts is ~70% (6–8), which is lower than that for eggs. It is difficult to avoid foods containing peanuts and hazelnuts. Patients are frequently informed that peanuts and hazelnuts pose high risk of anaphylaxis due to accidental consumption. Therefore, increased anxiety among patients and their families about consuming these foods after an OFC reportedly reduces reintroduction rates (6). In contrast, because the risk of anaphylaxis is lower with eggs than with nuts (13), children and their caregivers have lower anxiety levels and are more likely to reintroduce eggs at home after an OFC.

However, this study suggests that severe anxiety among parents about feeding eggs to their children after an OFC is the most important factor inhibiting egg reintroduction. Here, we evaluated caregiver anxiety using the ratio of post-OFC to pre-OFC anxiety. In other words, when a caregiver experiences more intense anxiety after the OFC than that before OFC, reintroducing eggs becomes difficult. Nevertheless, six caregivers (60%) reported a preference problem as to why children did not reintroduce eggs after OFC. This preference problem may have exacerbated the caregiver's anxiety, further preventing egg reintroduction in children.

Anxiety is broadly divided into state anxiety and trait anxiety, as evaluated by the STAI (State Trait Anxiety Inventory) (14). State anxiety refers to anxiety that is felt at specific times or situations. For example, when a child eats eggs after an OFC, the possibility of allergic symptoms causes the caregiver stress, which creates temporary anxiety. Trait anxiety is associated with constantly trying to avoid danger or having a tendency to worry. It has been reported that after an OFC, caregiver's state anxiety is reduced but not trait anxiety (15). Therefore, even if egg consumption is confirmed in an egg OFC, a caregiver with severe anxiety still may not want to feed their child eggs. Caregivers with severe trait anxiety may need more thoughtful and precise dietary guidance when reintroducing eggs at home after a step 3 OFC.

In a study that evaluated the anxiety of caregivers after OFC by STAI and evaluated the relationship with eating habits, it was reported that the mother's fear and anxiety negatively correlated with eating habits after OFC (9). If caregivers have severe anxiety after an egg OFC, they may not follow the dietary guidance given by healthcare providers. A psychological intervention to reduce anxiety among caregivers of children with FA was found to do so after 6 weeks (16). This indicates that when providing dietary guidance following an OFC, it is important to confirm at an early stage whether the caregiver is feeling more anxiety after an OFC than prior to the OFC. If they are, proactive psychological interventions may help increase the egg reintroduction rate.

In addition to caregiver anxiety, our results suggest that a history of anaphylaxis from eating eggs can make reintroduction difficult. It has been reported that caregivers with children who have FAs or a history of anaphylaxis experience a lower quality of life and increased levels of anxiety and stress (17, 18). Therefore, caution is warranted if there is a history of anaphylaxis from eggs, as caregivers may have more severe anxiety, reducing the reintroduction rate.

Furthermore, our results suggest that being older at the first OFC is associated with not reintroducing eggs. A study of age at OFC and food reintroduction rates after OFC found that 44 of 68 study participants (64.7%) were aged 0–4 years, whereas 27 of 57 (47.4%) were aged 9 or older, and, similar to the results of this study, lower reintroduction rates among older children were observed (6). Moreover, reintroduction rates have been reported to be lower when the food exclusion period prior to the OFC is longer than 2 years compared to when it is <2 years (8). These findings indicate that performing an OFC early, granted the right conditions, could help increase post-OFC reintroduction rates.

Additionally, our results suggest that the occurrence of allergic symptoms when eating eggs at home after an OFC is associated with not reintroducing eggs. Eating more egg protein amount than in the OFC or eating undercooked egg dishes is more likely to induce allergic symptoms than egg whites boiled for 20 min, as the food load here, and is more likely to induce allergic symptoms at home following an OFC. Therefore, when providing dietary guidance after an OFC, it is important to explain how symptoms can be induced and how to address them.

However, the allergic symptoms identified here did not require hospital visits and were not treated, suggesting they were mild. When a decision on the OFC result was deferred because the subjects had only mild abdominal pain, single coughs, or local skin symptoms, without allergic symptoms involving multiple organs, and were thus impossible to tell whether these were apparently due to FAs, 79.7% were negative when the allergen was eaten at home. The children who had symptoms exhibited only mild ones (19).

Therefore, the symptoms that occurred at home in this study were not necessarily allergic reactions to eating eggs. Following an OFC, explanations need to be given on foods that are more allergic than the load food and how to eat them, as well as on specific examples of allergic symptoms and how to deal with them depending on the severity. This could help increase reintroduction rates after OFC.

In summary, most children could reintroduce eggs in their diet a year or more after step 3 egg OFC. However, some factors require special attention as they indicate a higher risk of being unable to reintroduce eggs—including severe anxiety among caregivers following the OFC, the occurrence of allergic symptoms from eating eggs at home following the OFC, a history of anaphylaxis, and older age at the first egg OFC.

This study has three limitations. First, anxiety before and after the OFC was measured using a 10-point scale on the questionnaire, answered by caregivers. At present, no scientifically validated scale has been used to assess the association between FA and anxiety, and this method was used. However, from the results of studies that clarified the degree of anxiety using other STAIs, it is possible that the measurement method used in this study was not appropriate. Furthermore, since the anxiety before and after OFC was evaluated by the questionnaire conducted more than a year after OFC completion, there was a possibility that recall bias had occurred.

Second, with no previous research on egg reintroduction rates in Japan, we had no means of setting the sample size. The small sample size may have affected the results of the analysis. With this in mind, increasing the sample should be a task to consider in the future to provide more reliable results.

Finally, the dietary guidance given after an OFC differed after each test and may not have been consistent. Differences in interventions aimed at eating eggs after the OFC may have affected egg reintroduction. Therefore, a method for providing dietary guidance following an OFC must be established in the future. Therefore, as a dietary guidance method after OFC, it is a future task to create pamphlets and videos to establish unified dietary guidance.

## Impact Statement

This study was the first in Japan to investigate whether children were able to continue eating an egg at home following an OFC. The results showed that most of the children were able to eat eggs daily. Factors associated with difficulty in eating eggs included greater anxiety among post-test caregivers. For future medical care, importance should be given to regular monitoring of egg consumption status in affected children, and caregivers with severe anxiety should be provided with long-term outpatient follow-up and psychological interventions. We believe that following specific guidelines will enable children to consume eggs on a daily basis without worrying about OFC.

## Data Availability Statement

The raw data supporting the conclusions of this article will be made available by the authors, without undue reservation.

## Ethics Statement

The studies involving human participants were reviewed and approved by The Kindai University Faculty of Medicine and Kaizuka City Hospital. Written informed consent from the participants' legal guardian/next of kin was not required to participate in this study in accordance with the national legislation and the institutional requirements.

## Author Contributions

YT has made substantial contributions to the study's conception and design, data acquisition, analysis, and interpretation. TA, KY, and MN have made substantial contributions to the acquisition of data. NI and KS have been involved in drafting and revision of the manuscript for important intellectual content. All authors have read and approved the final draft of the manuscript.

## Conflict of Interest

The authors declare that the research was conducted in the absence of any commercial or financial relationships that could be construed as a potential conflict of interest.
